# K_1+2*x*_Ni_1−*x*_Fe_2_(AsO_4_)_3_ (*x* = 0,125): un nouvel arséniate à structure de type α-CrPO_4_

**DOI:** 10.1107/S2056989017000950

**Published:** 2017-01-24

**Authors:** Ridha Ben Smail, Mohamed Faouzi Zid

**Affiliations:** a Université de Tunis El Manar, Faculté des Sciences de Tunis, Laboratoire de Matériaux, Cristallochimie et Thermodynamique Appliquée, 2092 El Manar II, Tunis, Tunisia; b Université de Carthage, Institut Préparatoire aux Etudes d’Ingénieurs de Nabeul, Campus Universitaire, 8000 Mrazka, Nabeul, Tunisia

**Keywords:** crystal structure, CHARDI, arsenate, BVS, α-CrPO_4_, Madelung energy

## Abstract

A new arsenate K_1+2*x*_Ni_1−*x*_Fe_2_(AsO_4_)_3_ (*x* = 1/8) {potassium nickel diiron(III) tris­[arsenate(V)]} was synthesized using a flux method and its crystal structure was determined from single-crystal X-ray diffraction data. This material was also characterized by qualitative energy dispersive X-ray spectroscopy (EDS) analysis. The crystal structure belongs to the α-CrPO_4_-structure type, space group *Imma*. It consists of a three-dimensional-framework built up from FeO_6_ and Ni_0.875_□_1.25_O_6_-octa­hedra and AsO_4_-tetra­hedra that are sharing corners and/or edges, generating tunnels running along the [010] and [001] directions in which the potassium cations are located. The proposed structural model was validated by bond-valence-sum calculations, charge-distribution (CHARDI) and Madelung energy analyses.

## Abstract 2

A new arsenate K_1 + 2*x*_Ni_1 - *x*_Fe_2_(AsO_4_)_3_ (*x* = 1/8) was synthesized using a flux method and its crystal structure was determined from single-crystal X-ray diffraction data. It appears closely related to the *α-*CrPO_4_ structure type. The proposed structural model was validated by bond-valence-sum calculations, charge-distribution and Madelung energy analyses

## Contexte chimique   

Les phosphates et les arséniates mixtes de métaux de transition et de métaux alcalins présentant des charpentes à tunnels ou des structures en couches constituent un champ prometteur pour diverses applications, notamment dans le domaine du stockage d’énergie comme des matériaux électro-actifs pour les batteries rechargeables au sodium ou lithium (Masquelier *et al.*, 1998 ▸; Arroyo-de Dompablo *et al.*, 2006 ▸; Nose *et al.*, 2013 ▸; Barpanda *et al.*, 2013 ▸; Essehli *et al.*, 2015 ▸); ces matériaux sont utilisés aussi comme échangeurs ioniques (De la Rochère *et al.*, 1985 ▸; Buckley *et al.*, 1987 ▸).

Dans ce contexte, de nombreux arséniates doubles de nickel et de métaux alcalins ont été étudiés jusqu’à présent et une grande diversité de structures a été observée: NaNiAsO_4_ (Jones *et al.*, 1987 ▸) et KNiAsO_4_ (Buckley *et al.*, 1988 ▸) deux arséniates isotypes à structures en couches apparentées à celle de Mica (Beneke & Lagaly, 1982 ▸), LiNiAsO_4_ (Mesa *et al.*, 1998 ▸) type olivine (Bragg & Brown, 1926 ▸), K_4_Ni_7_(AsO_4_)_6_ (Ben Smail *et al.*, 1999 ▸) et Na_4_Ni_7_(AsO_4_)_6_ (Ben Smail *et al.*, 2007 ▸), deux arséniates qui présentent des structures à tunnels de type β-xenophyllite (Marzouki *et al.*, 2013 ▸), Na_4_Ni_5_[(As_0,73_,P_0,27_)O_4_]_2_[(As_0,59_,P_1,41_)O_7_]_2_ (Ben Smail & Jouini, 2004 ▸), isostructural du phosphate Na_4_Ni_5_(PO_4_)_2_(P_2_O_7_)_2_ (Sanz *et al.*, 1999 ▸), Na_3_Ni_2_(As_0,1_,P_0,9_)O_4_(As_1,3_,P_0,7_)O_7_ et sa limite arséniate qui s’avère un conducteur ionique moyen (Ben Smail & Jouini, 2005 ▸), NaNi_4_(AsO_4_)_3_ (Ben Smail *et al.*, 2002 ▸), le premier arséniate de nickel à structure non-centrosymétrique, et LiNi_2_As_3_O_10_ le premier triarséniate de nickel présentant une structure à tunnels (Ben Smail & Zid, 2017*a* ▸).

La richesse structurale de ces arséniates nous a encouragés a‘ explorer le système quaternaire K_2_O–Fe_2_O_3_–NiO–As_2_O_5_. Cette investigation nous a permis d’isoler une nouvelle phase de composition chimique K_1+2*x*_Ni_1−*x*_Fe_2_(AsO_4_)_3_ (*x* = 0,125) (I) (Ben Smail & Driss, 2007 ▸), la seule à notre connaissance dans son système, de type structural α-CrPO_4_ (Glaum *et al.*, 1986 ▸). Le modèle structural proposé, particulièrement l’occupation partielle des sites K^+^ (4*e*) et Ni^2+^ (4*b*), a été confirmé par les trois modèles de validation suivants: la somme des valences de liaisons BVS (Brown, 2002 ▸; Adams, 2003 ▸), la méthode de distribution de charge CHARDI (Nespolo *et al.*, 2001 ▸; Nespolo, 2015 ▸, 2016 ▸; Nespolo & Guillot, 2016 ▸) et l’énergie de Madelung (Hoppe, 1966 ▸, 1970 ▸; Momma & Izumi, 2008 ▸).

## Analyse structurale   

**Figure 1:**
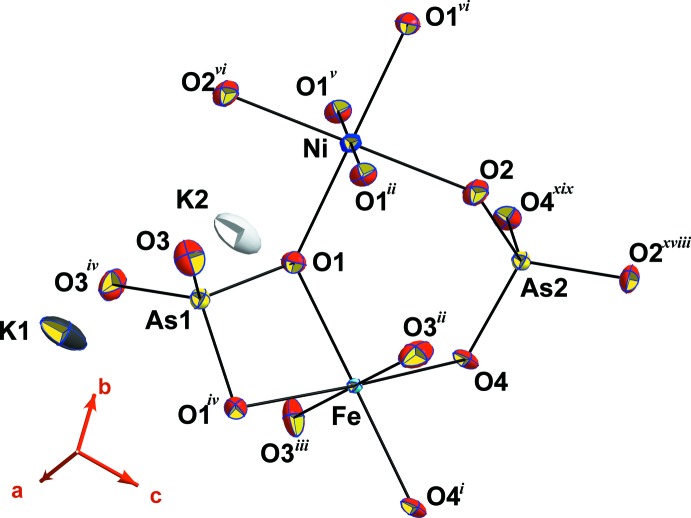
Polyèdres de coordination dans l’unité asymétrique de K_1,25_Ni_0,875_Fe_2_(AsO_4_)_3_. Les ellipsoïdes ont été définis avec 70% de probabilité. Voir le tableau 2 ▸ pour les codes de symétrie.

L’unité asymétrique de (I) contient quatre sites anioniques (8*h*), (8*i*) et deux (16*j*) occupés respectivement chacun par un anion O^2−^ et six sites cationiques parmi lesquels quatre sont complètement occupés respectivement par Fe^3+^ (8*g*), deux As^5+^ (8*g* et 4*e*) et K^+^ (*4e*) et les deux restants K^+^ (*4e*) et Ni^2+^ (*4b*) sont partiellement occupés (Fig. 1 ▸).

**Figure 2:**
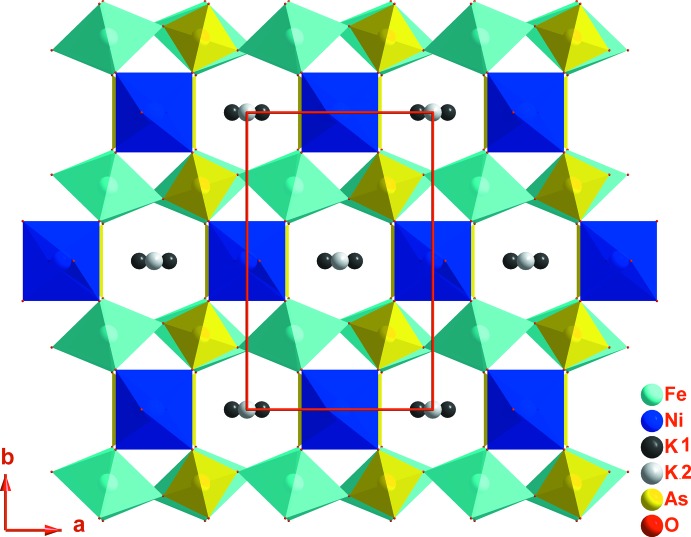
Projection, selon [010], de la structure de K_1,25_Ni_0,875_Fe_2_(AsO_4_)_3_.

**Figure 3:**
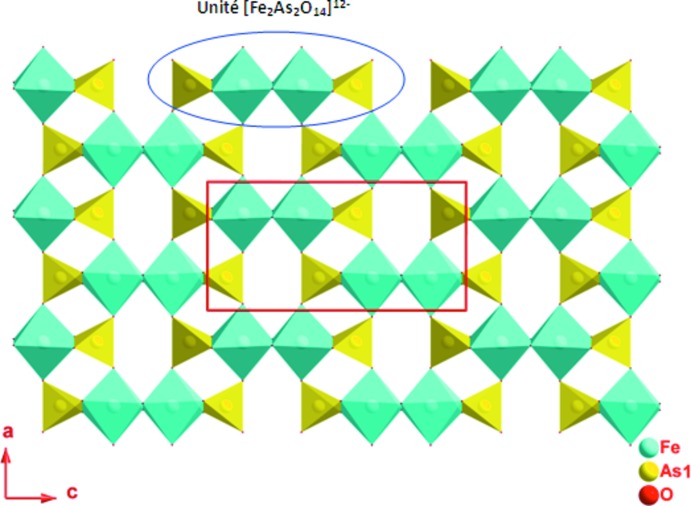
Projection d’une couche de la structure de K_1,25_Ni_0,875_Fe_2_(AsO_4_)_3_ selon la direction [010] mettant en jeux les unités [Fe_2_As_2_O_14_]^12−^.

**Figure 4:**
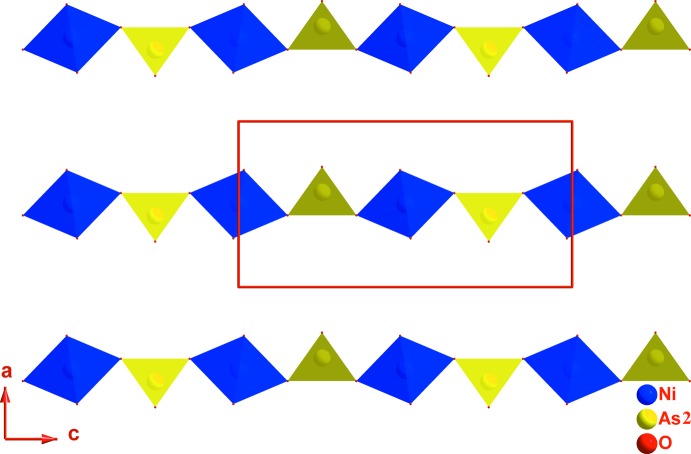
Disposition, selon la direction [001], des chaînes infinies [NiAsO_8_]^9−^_∞_ dans la structure de K_1,25_Ni_0,875_Fe_2_(AsO_4_)_3_.

La charpente anionique tridimensionnelle de (I) peut être décomposée en couches, parallèles au plan (010), et chaînes, parallèles à la direction [001]; ces deux composantes sont liées entre elles par des ponts mixtes de type Ni–O–As et Fe–O–Ni (Fig. 2 ▸). Dans une couche, deux octa­èdres FeO_6_ symétriques par un miroir et partageant une arête mettent chacun une arête commune avec un tétraèdre As1O_4_ pour former une unité linéaire centrosymétrique de formulation [Fe_2_As_2_O_14_]^12−^. Chaque unité est liée à chacune de ses quatre voisines par deux ponts Fe–O3–As1 (Fig. 3 ▸). Entre ces couches apparaissent les chaînes infinies [NiAsO_8_]^9−^_∞_ formées par les octa­èdres Ni_0,875_□_1,25_O_6_ et les tétraèdres As2O_4_ qui se lient par partage de sommets d’oxygène (Fig. 4 ▸). L’ossature tri-dimensionnelle engendrée par ce mode de connexion entre les tétraèdres et les octa­èdres fait apparaître des canaux parallèles aux directions [010] et [001] dans lesquels les cations K^+^ occupent deux sites distincts.

**Figure 5:**
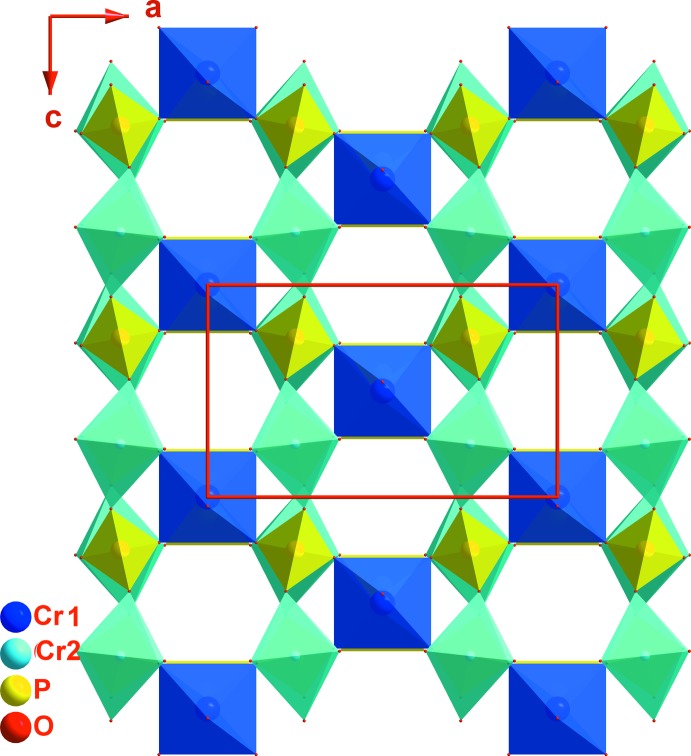
Projection de la structure du composé α-CrPO_4_ selon la direction [010].

Une recherche bibliographique a été effectuée sur la famille de composés de type α-CrPO_4_. On dénombre essentiellement seize monophosphates et deux monoarséniates (tableau 1 ▸). La comparaison des structures de ces composés à celle du α-CrPO_4_ montre que les deux sites octa­èdriques cristallographiquement indépendants Cr1 (4*a*) et Cr2 (8*g*) (Fig. 5 ▸ et 6 ▸) peuvent être occupés de différentes manières (tableau 1 ▸): tous les deux exclusivement par des cations trivalents (Rittner & Glaum, 1994 ▸; Attfield *et al.*, 1989 ▸), par une distribution statistique de cations di- et trivalents (Yahia *et al.*, 2016 ▸; Souiwa *et al.*, 2015*b* ▸; Essehli *et al.*, 2015 ▸), l’un par une répartition statistique de cations di- et trivalents et l’autre uniquement par des cations di- ou trivalents (Souiwa *et al.*, 2015*a* ▸; Ben Smail & Zid, 2017*b* ▸) ou l’un par des cations divalents et l’autre par des cations trivalents (Bouraima *et al.*, 2016 ▸; Ouaatta *et al.*, 2015 ▸; Alhakmi *et al.*, 2013*a* ▸,*b* ▸; Assani *et al.*, 2013 ▸; Korzenski *et al.*, 1999 ▸; Hidouri *et al.*, 2004 ▸; Kinomura *et al.*, 1989 ▸). La dernière répartition cationique est observée dans l’arséniate objet de ce travail.

Ces matériaux diffèrent aussi par la répartition des cations alcalins et alcalino-terreux dans les deux types de sites (*4e*) et (*4b*) ou (*4a*) se trouvant dans les canaux formés par la charpente covalente. En effet, dans le cas des arséniates K_1,25_Ni_0,875_Fe_2_(AsO_4_)_3_ et NaCa_0,77_Ni_2,54_Al_0,46_(AsO_4_)_3_ et les phosphates Na_1,28_Ni_0,86_Fe_2_(PO_4_)_3_, Na_1,2_Mg_1,2_Cr_1,8_(PO_4_)_3_, α-Na_2_Ni_2_Fe(PO_4_)_3_ et NaCoCr_2_(PO_4_)_3_, et Na_2_Ni_2_Cr(PO_4_)_3_ les cations alcalins sont répartis sur ces deux types de sites. Ces sites sont totalement vides dans la structure de α-CrAsO_4_, RhPO_4_ et α-CrPO_4_ (Figs. 5 ▸ et 6) alors qu’un seul, (*4e*), corres­pondant à l’atome K1 dans le cas de (I) est occupé par le sodium dans NaV^II^V^III^_2_(PO_4_)_3_, NaCoCr_2_(PO_4_)_3_ et NaNiCr_2_(PO_4_)_3_, par le strontium dans SrFe^II^_2_Fe^III^(PO_4_)_3_, SrMn^II^_2_Mn^III^(PO_4_)_3_, SrCo_2_Fe(PO_4_)_3_ et SrNi_2_Fe(PO_4_)_3_, par le plomb dans PbMn^II^_2_Mn^III^(PO_4_)_3_ et par le baryum dans BaMn^II^_2_Mn^III^(PO_4_)_3_. Cette comparaison montre que la position (*4e*) située à l’inter­section des deux types de canaux de la charpente de α-CrPO_4_ est la plus favorable pour accueillir les cations alcalins et/ou alcalinoterreux.

La comparaison de la structure de l’arséniate étudié ici avec celle du phosphate Na_1,28_Ni_0,86_Fe_2_(PO_4_)_3_ révèle une différence nette au niveau de la coordination des atomes alcalins. En effet, dans une sphère de coordination de rayon 3 Å, les deux atomes de potassium dans l’arséniate sont octa­coordinés (K1: 4 + 2 + 2 et K2: 4 + 4) alors que dans le phosphate les deux atomes de sodium sont hepta­coordinés (Na1: 4 + 2 + 1 et Na2: 4 + 3).

L’examen des longueurs des liaisons Ni/Fe/As—O et O—O et des valeurs des angles de liaisons O—Ni/Fe/As—O dans les octa­èdres Ni_0,875_□_0,125_O_6_ et FeO_6_ et les tétraèdres AsO_4_ dans la structure de (I) montre que ces derniers sont irréguliers (tableau 2 ▸). Pour évaluer leur distorsion, nous les avons examinés à la fois par le calcul de leur nombres de coordination effectifs ECoNs (abréviation de l’anglais ‘**E**ffective **Co**ordination **N**umber**s**’) (Nespolo, 2015 ▸; Nespolo *et al.*, 2001 ▸) ainsi que leurs indices de distorsion (ID) moyennant les formules de Baur (1974 ▸) et Wildner (1992 ▸) définies comme suit: 
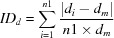


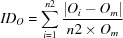


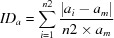



où *d* est la distance Ni/Fe/As—O, O est la distance O—O (entre les atomes d’oxygène voisins), a est l’angle de liaison O—Ni/Fe/As—O, l’indice *i* indique les valeurs individuels, l’indice *m* correspond à la valeur moyenne dans chaque polyèdre, *n*1 = 4 et *n*2 = 6 pour un tétraèdre et *n*1 = 6 et *n*2 = 12 pour un octa­èdre.

Les valeurs obtenues sont résumées au tableau 3 ▸. Ces paramètres de distorsion montrent que le tétraèdre As1O_4_ est légèrement plus distordu que As2O_4_: As1 (ECoN = 3,94 et 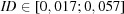) et As2 (ECoN = 3,99 et 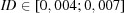) . Ceci est dû au fait que dans la structure les tétraèdres As2O_4_ ne partagent que des sommets avec les autres polyèdres alors que les tétraèdres As2O_4_ partagent à la fois des sommets et des arêtes. En revanche, les octa­èdres FeO_6_ et Ni_0,875_□_0,125_O_6_ sont relativement réguliers et présentent des facteurs de distorsion assez comparables: ECoN = 5,99 et 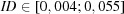.

L′ECoN du cation K1 correspond bien à la valeur idéale (CN = 8) puisque le polyèdre est régulier. Le polyèdre de coordination de K2 est assez distordu avec un ECoN = 5,72 qui s’écarte du nombre de coordination classique CN = 8.

## Calcul de l′énergie de Madelung   

La principale contribution à l’énergie de liaison des cristaux ioniques est d’origine électrostatique; on l’appelle énergie de Madelung.

Différentes méthodes ont été proposées pour calculer cette énergie coulombienne. Il est possible d’y discerner deux grands groupes de résolution: certains remplacent les ions par des distributions de charge continues, d’autres gardent le caractère discret des charges ponctuelles pour aboutir à la valeur de l’énergie de Madelung par des méthodes directes.

Dans notre cas, cette énergie a été déterminée par la méthode de Fourier (Harris & Monkhorst, 1970 ▸), employant une distribution continue de charges, moyennant l’algorithme MADEL incorporé dans le programme *VESTA 3* (Momma & Izumi, 2008 ▸).

La sommation dans le réseau direct a été faite en utilisant une sphère ionique de rayon 1,2 Å. Celle dans le réseau réciproque a été effectuée avec des coefficients de Fourier allant jusqu’à 4 Å.

La valeur de l’énergie de Madelung du composé (I), calculée à partir des données cristallographiques obtenues par diffraction des RX, est égale à −82933,6 kJ mol^−1^. Cette valeur est en bon accord avec la somme des énergies de Madelung des oxydes binaires (tableau 4 ▸) correspondant à la composition chimique: K_1,25_Ni_0,875_Fe_2_(AsO_4_)_3_ = 0,625 K_2_O + 0,875 NiO + Fe_2_O_3_ + 1,5 As_2_O_5_; −83156,301 kJ mol^−1^. Le faible écart entre ces deux valeurs (déviation 0,27%) confirme la validité du modèle structural proposé.

Nous avons ainsi vérifié la convergence des valeurs de l’énergie de Madelung en faisant varier les deux paramètres: rayon de la sphère ionique et la taille du domaine de l’espace réciproque signalés précédemment.

## Synthèse et caractérisation   

La croissance des cristaux du composé K_1,25_Ni_0,875_Fe_2_(AsO_4_)_3_ a été effectuée dans un flux de K_2_Mo_2_O_7_. Les réactifs, K_2_CO_3_ (0,138 g, Prolabo), Ni(NO_3_)_2_·6H_2_O (0,58 g, Prolabo), Fe(NO_3_)_3_·9H_2_O (0,81g, Prolabo), NH_4_H_2_AsO_4_ (0,64g, préparé au laboratoire, ASTM 01–775) et (NH_4_)_6_Mo_7_O_24_·4H_2_O (1,412 g, Fluka) ont été finement broyés dans un mortier en agate. Le mélange obtenu, a été transvasé dans creuset en porcelaine puis préchauffé dans un four à moufle à 623 K durant 12 heures en vue d’éliminer les composés volatils. Après refroidissement et un broyage d’homogénéisation poussé, le produit obtenu a été chauffé progressivement jusqu’à 1073 K, température de fusion du mélange réactionnel, cette température a été maintenue pendant deux heures afin de favoriser la germination et la croissance des cristaux. Par la suite, il a subi un refroidissement lent avec une vitesse de 5 K h^-1^ jusqu’à 873 K puis le four a été éteint. Des cristaux de couleur brune et de taille suffisante pour une étude structurale par diffraction des rayons X ont été séparés du flux par des lavages successifs à l’eau bouillante.

**Figure 7:**
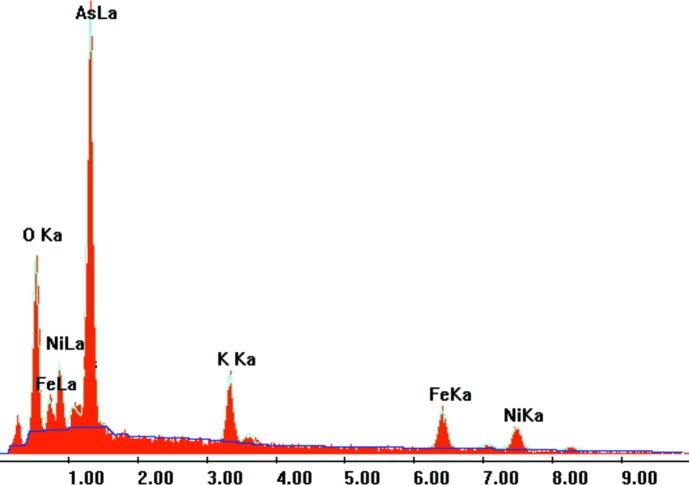
Analyse qualitative EDS d’un cristal du composé K_1,25_Ni_0,875_Fe_2_(AsO_4_)_3_.

L’analyse qualitative par spectroscopie à rayons X à dispersion d’énergie EDS (abréviation de l’anglais ‘**E**nergy **D**ispersive X-ray **S**pectroscopy’) sur microscope électronique à balayage de type JEOL-JSM-5400 de quelques cristaux a confirmé la présence de tous les éléments chimiques attendus (Fig. 7 ▸).

## Affinement   

Les données cristallographiques, les conditions de la collecte et les résultats de l’affinement de la structure de (I) sont résumées au tableau 5 ▸. La structure a été affinée dans le groupe d’espace non conventionnel *Ibmm*.

Les deux sites K^+^ (4*e*) et Ni^2+^ (4*b*) ont été affinés au début comme étant totalement occupés, cependant plusieurs anomalies ont été observées:

(i) la neutralité électrique du composé n’est pas vérifiée,

(ii) les facteurs de reliabilité sont très élevés (*R* = 5,42% et *wR* = 16,4%),

(iii) les densités électroniques résiduelles sont assez grandes (Δρ_max_ = 5,42 e Å^−3^ et Δρ_min_ = −3,57 e Å^−3^) et elles sont situées respectivement à 0,00 Å et 0,88 Å de K^+^ (4*e*).

(iv) les facteurs d’agitation thermiques isotropes de K^+^ (4*e*) et Ni^2+^ (4*b*) sont anormalement élevés.

Dans une deuxième étape, l’affinement a porté sur les occupations de ces deux sites qui ont dévié de l’occupation totale [K^+^ (4*e*) = 0,25 (3) et Ni^2+^ (4*b*) = 0,875 (3)]. Les conditions de la neutralité électrique pour ce composé nous ont encouragées à fixer ces derniers à 25% pour le K^+^ (4*e*) et 87,5% pour le Ni^2+^ (4*b*). Les valeurs de charges calculées *Q* (Nespolo *et al.*, 2001 ▸; Nespolo, 2015 ▸, 2016 ▸; Nespolo & Guillot, 2016 ▸) et des valences *V* (Brown, 2002 ▸; Adams, 2003 ▸) pondérées par les taux d’occupation sont en bon accord avec les degrés d’oxydation pondérés par les taux d’occupation (tableau 6 ▸). L’écart absolu moyen en pourcentage MAPD (Nespolo, 2016 ▸) (abréviation de l’anglais ‘**M**ean **A**bsolute **P**ercentage **D**eviation’) qui mesure l’accord entre les nombres d’oxydation formel (*q*) et les charges calculées (*Q*), est égal à 2,9%.

L’affinement final des taux d’occupation des sites (4*b*) et (4*e*) occupés respectivement par les cations Ni^2+^ et K^+^ a réduit énormément les facteurs de reliabilté et a amélioré les facteurs d’agitation thermique.

Les densités électroniques résiduelles maximale et minimale dans la Fourier-différence finale sont acceptables et sont situées respectivement à 0,55 Å de Fe et à 1,29 Å de O1.

## Supplementary Material

Crystal structure: contains datablock(s) I. DOI: 10.1107/S2056989017000950/vn2121sup1.cif

Structure factors: contains datablock(s) I. DOI: 10.1107/S2056989017000950/vn2121Isup2.hkl

CCDC reference: 1528356

Additional supporting information:  crystallographic information; 3D view; checkCIF report

## Figures and Tables

**Figure 6:**
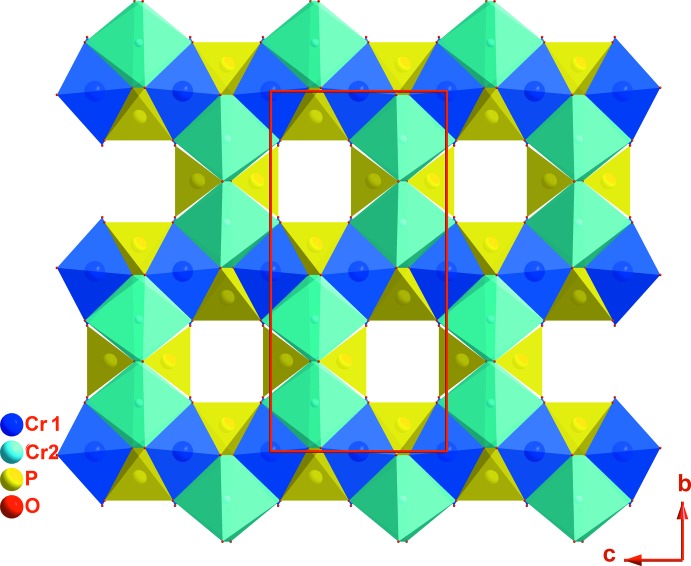
Projection de la structure du composé α-CrPO_4_ selon la direction [100].

**Table 1:** Caractéristiques cristallographiques (Å) des phosphates et arséniates à structure type α-CrPO_4_ rencontrés dans la littérature et répartition des cations sur les deux sites octa­èdriques Cr1 (*4a*) et Cr2 (*8g*)

Composé	groupe d’éspace	*a*	*b*	*c*	méthode de diffraction	Cr1 (*4a*)	Cr2 (*8g*)
α-CrPO_4_ *^*a*^*	*Imma*	10,403 (2)	12,898 (2)	6,299 (1)	DRXM	Cr^3+^	Cr^3+^
RhPO_4_ *^*b*^*	*Imma*	10,391	13,091	6,391	DRXP	Rh^3+^	Rh^3+^
α-CrAsO_4_ *^*c*^*	*Imma*	10,5466 (1)	13,2424 (1)	6,4612 (1)	DNP	Cr^3+^	Cr^3+^
NaNiCr_2_(PO_4_)_3_ *^*d*^*	*Imma*	10,3988 (3)	12,9858 (6)	6,3634 (3)	DNP	0,223 (7) Ni^2+^ + 0,777 (7) Cr^3+^	0,388 (3) Ni^2+^ + 0,612 (3) Cr^3+^
Na_2_Ni_2_Cr(PO_4_)_3_ *^*d*^*	*Imma*	10,3667 (4)	13,0988 (7)	6,4817 (3)	DNP	0,358 (10) Ni^2+^ + 0,642 (10) Cr^3+^	0,821 (5) Ni^2+^ + 0,179 (5) Cr^3+^
NaMgCr_2_(PO_4_)_3_ *^*e*^*	*Imma*	10,406 (1)	12,998 (1)	6,363 (1)	DRXM	0,812 Cr^3+^ + 0,188 Mg^2+^	0,594 Cr^3+^ + 0,406 Mg^2+^
Na_1,2_Mg_1,2_Cr_1,8_(PO_4_)_3_ *^*e*^*	*Imma*	10,393 (1)	13,030 (1)	6,394 (4)	DRXM	0,845 Cr^3+^ + 0,155 Mg^2+^	0,477 Cr^3+^ + 0,523 Mg^2+^
NaZnCr_2_(PO_4_)_3_ *^*e*^*	*Imma*	10.412 (1)	13,026 (1)	6,377 (1)	DRXM	0,828 Cr^3+^ + 0,172 Zn^2+^	0,586 Cr^3+^ + 0,414 Zn^2+^
α-Na_2_Ni_2_Fe(PO_4_)_3_ *^*f*^*	*Imma*	10,42821 (12)	13,19862 (15)	6,47634 (8)	DRXP	0,5 Ni^2+^ + 0,5 Fe^3+^	0,75 Ni^2+^ + 0,25 Fe^3+^
NaCoCr_2_(PO_4_)_3_ *^*g*^*	*Imma*	10,413 (1)	13,027 (1)	6,372 (1)	DRXP	Cr^3+^	0,5 Cr^3+^ + 0,5 Co^2+^
NaCa_0,77_Ni_2,54_Al_0,46_(AsO_4_)_3_ *^*h*^*	*Imma*	10,419 (2)	13,496 (2)	6,669 (2)	DRXM	0,54 (1) Ni^2+^ + 0,46 (1) Al^3+^	Ni^2+^
SrCo_2_Fe(PO_4_)_3_^*i*^	*Imma*	10,4097 (2)	13,2714 (3)	6,5481 (2)	DRXM	Fe^3+^	Co^2+^
SrNi_2_Fe(PO_4_)_3_ *^*j*^*	*Imma*	10,3881 (11)	13,1593 (13)	6,5117 (7)	DRXM	Fe^3+^	Ni^2+^
PbMn^II^_2_Mn^III^(PO_4_)_3_ *^*k*^*	*Imma*	10,2327 (8)	13,9389 (9)	6,6567 (4)	DRXM	Mn^3+^	Mn^2+^
SrMn^II^_2_Mn^III^(PO_4_)_3_ *^*l*^*	*Imma*	10,2373 (10)	13,8981 (15)	6,6230 (6)	DRXM	Mn^3+^	Mn^2+^
BaMn^II^_2_Mn^III^(PO_4_)_3_ *^*m*^*	*Imma*	10,3038 (7)	14,0163 (11)	6,7126 (4)	DRXM	Mn^3+^	Mn^2+^
SrFe^II^_2_Fe^III^(PO_4_)_3_ *^*n*^*	*Imma*	10,452 (3)	13,429 (3)	6,528 (2)	DRXM	Fe^3+^	Fe^2+^
Na_1,28_Ni_0,86_Fe_2_(PO_4_)_3_ *^*o*^*	*Ibmm*	6,438 (2)	10,515 (3)	13,166 (3)	DRXM	0,86 Ni^2+^ + 0,14 □	Fe^3+^
NaV^II^V^III^_2_(PO_4_)_3_ *^*p*^*	*Imma*	10,488 (2)	13,213 (3)	6,455 (1)	DRXM	V^3+^	V^2+^
K_1,25_Ni_0,875_Fe_2_(AsO_4_)_3_ *^*q*^*	*Ibmm*	6,737 (2)	10,773 (3)	13,574 (3)	DRXM	0,875 Ni^2+^ + 0,125 □	Fe^3+^

Notes: DNP = diffraction des neutrons sur poudre; DRXM = diffraction des rayons X sur moncristal; DRXP = diffraction des rayons X sur poudre; □ = site vacant. Références: (*a*) Glaum *et al.* (1986 ▸); (*b*) Rittner & Glaum (1994 ▸); (*c*) Attfield *et al.* (1989 ▸); (*d*) Yahia *et al.* (2016 ▸); (*e*) Souiwa *et al.* (2015*b* ▸); (*f*) Essehli *et al.* (2015 ▸); (*g*) Souiwa *et al.* (2015*a* ▸); (*h*) Ben Smail & Zid (2017*b* ▸); (*i*) Bouraima *et al.* (2016 ▸); (*j*) Ouaatta *et al.* (2015 ▸); (*k*) Alhakmi *et al.* (2013*a* ▸); (*l*) Alhakmi *et al.* (2013*b* ▸); (*m*) Assani *et al.* (2013 ▸); (*n*) Korzenski *et al.* (1999 ▸); (*o*) Hidouri *et al.* (2004 ▸); (*p*) Kinomura *et al.* (1989 ▸); (*q*) ce travail.

**Table 2:** Longueurs des liaisons sélectionnées (Å)

Fe—O4	2,051 (3)	K1—O2^xii^	2,759 (5)
Fe—O4^i^	2,051 (3)	K1—O2^xiii^	2,759 (5)
Fe—O3^ii^	2,069 (3)	K2—O1^xiv^	2,461 (3)
Fe—O3^iii^	2,069 (3)	K2—O1^v^	2,461 (3)
Fe—O1	2,077 (3)	K2—O1^xv^	2,461 (3)
Fe—O1^iv^	2,077 (3)	K2—O1	2,461 (3)
Ni—O1	2,017 (3)	K2—O3^xvi^	2,939 (4)
Ni—O1^v^	2,017 (3)	K2—O3^iv^	2,939 (4)
Ni—O1^ii^	2,017 (3)	K2—O3^xvii^	2,939 (4)
Ni—O1^vi^	2,017 (3)	K2—O3^iii^	2,939 (4)
Ni—O2	2,047 (5)	As1—O3^iv^	1,672 (3)
Ni—O2^vi^	2,047 (5)	As1—O3	1,672 (3)
K1—O3	2,740 (3)	As1—O1^iv^	1,740 (3)
K1—O3^vii^	2,740 (3)	As1—O1	1,740 (3)
K1—O3^viii^	2,740 (3)	As2—O2^xviii^	1,682 (4)
K1—O3^ix^	2,740 (3)	As2—O2	1,682 (4)
K1—O4^x^	2,745 (5)	As2—O4	1,706 (4)
K1—O4^xi^	2,745 (5)	As2—O4^xix^	1,706 (4)

Codes de symmétrie: (i) 
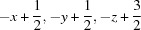
; (ii) 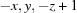; (iii) 
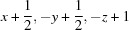
; (iv) 
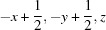
; (v) 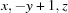; (vi) 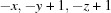; (vii) 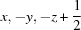; (viii) 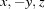; (ix) 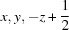; (x) 
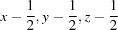
; (xi) 
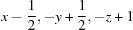
; (xii) 
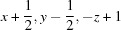
; (xiii) 
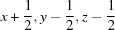
; (xiv) 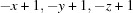; (xv) 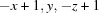; (xvi) 
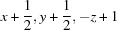
; (xvii) 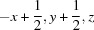; (xviii) 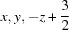; (xix) 
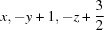
.

**Table 3:** Nombre de coordination effectif (ECoN) et indice de distorsion (ID) des polyèdres cationiques dans la structure de (I)

Cation	Fe	Ni	As1	As2
NC	6	6	4	4
ECoN	5,99	5,99	3,94	3,99
*d*_m_ (Å)	2,066	2,027	1,706	1,694
*a*_m_ (°)	90,03	90,00	109,19	109,43
*o*_m_ (Å)	2,916	2,866	2,774	2,765
*ID*_*d*_	0,004	0,006	0,020	0,007
*ID*_*a*_	0,055	0,027	0,057	0,007
*ID*_*o*_	0,043	0,021	0,017	0,004

Note: NC est le nombre de coordination classique.

**Table 4:** Energies de Madelung (*E*_M_ en kJ mol^−1^) des oxydes binaires K_2_O*^*a*^*, NiO*^*b*^*, Fe_2_O_3_ *^*c*^* et As_2_O_5_ *^*d*^* calculées à partir des données structurales correspondantes

Oxyde	K_2_O	NiO	Fe_2_O_3_	As_2_O_5_
*E*_M_	−2512,752	−4647,168	−16827,579	−40461,32

Notes: (*a*) Zintl *et al.* (1934 ▸); (*b*) Malingowski *et al.* (2012 ▸); (*c*) Zachariasen (1928 ▸); (*d*) Jansen (1978 ▸).

**Table 5:** Détails expérimentaux

Données cristallines	
Formule chimique	K_1.25_Ni_0.88_Fe_2_(AsO_4_)_3_
*M*_r_	628,71
Système cristallin, groupe d’espace	Orthorhombique, *I* *b* *m* *m*
Température (K)	298
*a*, *b*, *c* (Å)	6,737 (2), 10,773 (3), 13,574 (3)
*V* (Å^3^)	985,2 (5)
*Z*	4
Type de rayonnement	Mo *K*α
μ (mm^−1^)	15,16
Taille du cristal (mm)	0,25 × 0,18 × 0,07

Collecte de données	
Diffractomètre	Enraf–Nonius CAD-4
Correction d’absorption	ψ scan (North *et al.*, 1968 ▸)
*T*_min_, *T*_max_	0,372, 0,999
Nombre de réflexions mesurées, indépendantes et observées [*I* > 2σ(*I*)]	1323, 591, 548
*R*_int_	0,026
(sin θ/λ)_max_ (Å^−1^)	0,638

Affinement	
*R*[*F* ^2^ > 2σ(*F* ^2^)], *wR*(*F* ^2^), *S*	0,028, 0,089, 1,10
Nombre de réflexions	591
Nombre de paramètres	58
Δρ_max_, Δρ_min_ (e Å^−3^)	1,12, −0,92

Programmes informatiques: *CAD-4 EXPRESS* (Duisenberg, 1992 ▸; Macíček & Yordanov, 1992 ▸), *XCAD4* (Harms & Wocadlo, 1995 ▸), *SHELXS97* (Sheldrick, 2008 ▸), *SHELXL2014* (Sheldrick, 2015 ▸), *DIAMOND* (Brandenburg, 2006 ▸), *WinGX* (Farrugia, 2012 ▸) et *publCIF* (Westrip, 2010 ▸).

**Table 6:** Analyse BVS et CHARDI des polyèdres cationiques dans la structure de (I) (la structure est décrite comme étant un empilement de polyèdres centrés sur les cations)

Cation	*q*(*i*).sof(*i*)	*V*(*i*).sof(*i*)	*Q*(*i*)
Fe	3,00	2,65	2,85
Ni	1,76	1,89	1,90
K1	1,00	1,33	1,00
K2	0,25	0,44	0,26
As1	5,00	4,83	5,03
As2	5,00	4,90	5,09

Notes: *q* est le nombre d’oxydation formel; sof est le taux d’occupation du site (de l’anglais: site occupancy factor); *Q* est la charge calculée.

## supplementary crystallographic information

### Crystal data

**Table:**

K_1.25_Ni_0.88_Fe_2_(AsO_4_)_3_	*D*_x_ = 4.239 Mg m^−^^3^
*M**_r_* = 628.71	Mo *K*α radiation, λ = 0.71073 Å
Orthorhombic, *I**b**m**m*	Cell parameters from 25 reflections
*a* = 6.737 (2) Å	θ = 10.8–14.5°
*b* = 10.773 (3) Å	µ = 15.16 mm^−^^1^
*c* = 13.574 (3) Å	*T* = 298 K
*V* = 985.2 (5) Å^3^	Prism, brown
*Z* = 4	0.25 × 0.18 × 0.07 mm
*F*(000) = 1181	

### Data collection

**Table:**

Enraf–Nonius CAD-4 diffractometer	*R*_int_ = 0.026
ω/2θ scans	θ_max_ = 27.0°, θ_min_ = 3.0°
Absorption correction: ψ scan (North *et al.*, 1968)	*h* = −1→8
*T*_min_ = 0.372, *T*_max_ = 0.999	*k* = 0→13
1323 measured reflections	*l* = −17→17
591 independent reflections	2 standard reflections every 120 reflections
548 reflections with *I* > 2σ(*I*)	intensity decay: 1%

### Refinement

**Table:**

Refinement on *F*^2^	0 restraints
Least-squares matrix: full	*w* = 1/[σ^2^(*F*_o_^2^) + (0.0629*P*)^2^ + 3.0716*P*] where *P* = (*F*_o_^2^ + 2*F*_c_^2^)/3
*R*[*F*^2^ > 2σ(*F*^2^)] = 0.028	(Δ/σ)_max_ < 0.001
*wR*(*F*^2^) = 0.089	Δρ_max_ = 1.12 e Å^−^^3^
*S* = 1.10	Δρ_min_ = −0.92 e Å^−^^3^
591 reflections	Extinction correction: SHELXL2014 (Sheldrick, 2015), Fc^*^=kFc[1+0.001xFc^2^λ^3^/sin(2θ)]^-1/4^
58 parameters	Extinction coefficient: 0.0016 (3)

### Special details

**Table:**

Geometry. All esds (except the esd in the dihedral angle between two l.s. planes) are estimated using the full covariance matrix. The cell esds are taken into account individually in the estimation of esds in distances, angles and torsion angles; correlations between esds in cell parameters are only used when they are defined by crystal symmetry. An approximate (isotropic) treatment of cell esds is used for estimating esds involving l.s. planes.

### Fractional atomic coordinates and isotropic or equivalent isotropic displacement parameters (Å^2^)

**Table:**

	*x*	*y*	*z*	*U*_iso_*/*U*_eq_	Occ. (<1)
Fe	0.2500	0.2500	0.63616 (6)	0.0043 (3)	
Ni	0.0000	0.5000	0.5000	0.0067 (3)	0.875
K1	0.0797 (3)	0.0000	0.2500	0.0219 (6)	
K2	0.5000	0.5000	0.5000	0.027 (2)	0.25
As1	0.2500	0.2500	0.42905 (4)	0.0068 (3)	
As2	0.07167 (11)	0.5000	0.7500	0.0059 (3)	
O1	0.2062 (5)	0.3657 (3)	0.5158 (2)	0.0094 (6)	
O2	−0.0672 (6)	0.5000	0.6470 (3)	0.0105 (9)	
O3	0.0501 (5)	0.2095 (3)	0.3635 (2)	0.0152 (7)	
O4	0.2254 (6)	0.3742 (4)	0.7500	0.0093 (9)	

### Atomic displacement parameters (Å^2^)

**Table:**

	*U*^11^	*U*^22^	*U*^33^	*U*^12^	*U*^13^	*U*^23^
Fe	0.0061 (5)	0.0033 (5)	0.0034 (5)	0.0008 (2)	0.000	0.000
Ni	0.0090 (7)	0.0057 (6)	0.0054 (6)	0.000	−0.0010 (5)	0.000
K1	0.0096 (10)	0.0117 (9)	0.0445 (15)	0.000	0.000	0.000
K2	0.009 (4)	0.020 (4)	0.053 (7)	0.000	0.005 (4)	0.000
As1	0.0068 (4)	0.0076 (4)	0.0061 (4)	0.00005 (17)	0.000	0.000
As2	0.0062 (5)	0.0060 (4)	0.0055 (5)	0.000	0.000	0.000
O1	0.0108 (13)	0.0082 (13)	0.0091 (13)	0.0015 (13)	−0.0015 (11)	−0.0004 (12)
O2	0.011 (2)	0.014 (2)	0.007 (2)	0.000	−0.0029 (15)	0.000
O3	0.0102 (15)	0.0214 (18)	0.0141 (18)	−0.0003 (13)	−0.0040 (12)	−0.0054 (13)
O4	0.010 (2)	0.0054 (18)	0.012 (2)	0.0030 (15)	0.000	0.000

### Geometric parameters (Å, º)

**Table:**

Fe—O4	2.051 (3)	K1—K2^iv^	3.4357 (8)
Fe—O4^i^	2.051 (3)	K2—O1^xvii^	2.461 (3)
Fe—O3^ii^	2.069 (3)	K2—O1^vii^	2.461 (3)
Fe—O3^iii^	2.069 (3)	K2—O1^xviii^	2.461 (3)
Fe—O1	2.077 (3)	K2—O1	2.461 (3)
Fe—O1^iv^	2.077 (3)	K2—O3^xix^	2.939 (4)
Fe—As1	2.8113 (12)	K2—O3^iv^	2.939 (4)
Fe—K2^iv^	3.6751 (8)	K2—O3^xx^	2.939 (4)
Fe—K2	3.6751 (8)	K2—O3^iii^	2.939 (4)
Fe—K1^v^	3.8177 (13)	K2—As1^xvii^	3.3193 (7)
Fe—K1^vi^	3.8177 (13)	K2—As1^xx^	3.3193 (7)
Ni—O1	2.017 (3)	K2—As1^iii^	3.3193 (7)
Ni—O1^vii^	2.017 (3)	K2—As1	3.3193 (7)
Ni—O1^ii^	2.017 (3)	As1—O3^iv^	1.672 (3)
Ni—O1^viii^	2.017 (3)	As1—O3	1.672 (3)
Ni—O2	2.047 (5)	As1—O1^iv^	1.740 (3)
Ni—O2^viii^	2.047 (5)	As1—O1	1.740 (3)
Ni—K2^ix^	3.3685 (10)	As1—K2^iv^	3.3193 (7)
Ni—K2	3.3685 (10)	As1—K1^xxi^	3.8049 (9)
K1—O3	2.740 (3)	As2—O2^xxii^	1.682 (4)
K1—O3^x^	2.740 (3)	As2—O2	1.682 (4)
K1—O3^xi^	2.740 (3)	As2—O4	1.706 (4)
K1—O3^xii^	2.740 (3)	As2—O4^xxiii^	1.706 (4)
K1—O4^xiii^	2.745 (5)	As2—K1^xxiv^	3.314 (2)
K1—O4^xiv^	2.745 (5)	As2—K1^v^	3.423 (2)
K1—O2^xv^	2.759 (5)	O2—K1^xxiv^	2.759 (5)
K1—O2^xvi^	2.759 (5)	O3—Fe^xiv^	2.069 (3)
K1—As2^xvi^	3.314 (2)	O3—K2^iv^	2.939 (4)
K1—As2^xiii^	3.423 (2)	O4—Fe^i^	2.051 (3)
K1—K2^xiii^	3.4357 (8)	O4—K1^v^	2.745 (5)
			
O4—Fe—O4^i^	82.21 (17)	O2^xv^—K1—K2^iv^	68.55 (9)
O4—Fe—O3^ii^	93.27 (16)	O2^xvi^—K1—K2^iv^	129.43 (10)
O4^i^—Fe—O3^ii^	86.55 (15)	As2^xvi^—K1—K2^iv^	98.99 (3)
O4—Fe—O3^iii^	86.55 (15)	As2^xiii^—K1—K2^iv^	81.01 (3)
O4^i^—Fe—O3^iii^	93.27 (16)	K2^xiii^—K1—K2^iv^	162.02 (6)
O3^ii^—Fe—O3^iii^	179.77 (19)	O1^xvii^—K2—O1^vii^	108.00 (14)
O4—Fe—O1	100.94 (12)	O1^xvii^—K2—O1^xviii^	72.00 (14)
O4^i^—Fe—O1	175.01 (13)	O1^vii^—K2—O1^xviii^	180.0
O3^ii^—Fe—O1	89.40 (13)	O1^xvii^—K2—O1	180.0
O3^iii^—Fe—O1	90.79 (14)	O1^vii^—K2—O1	72.00 (14)
O4—Fe—O1^iv^	175.02 (14)	O1^xviii^—K2—O1	108.00 (14)
O4^i^—Fe—O1^iv^	100.94 (12)	O1^xvii^—K2—O3^xix^	60.73 (10)
O3^ii^—Fe—O1^iv^	90.79 (14)	O1^vii^—K2—O3^xix^	65.57 (10)
O3^iii^—Fe—O1^iv^	89.40 (13)	O1^xviii^—K2—O3^xix^	114.43 (10)
O1—Fe—O1^iv^	76.19 (17)	O1—K2—O3^xix^	119.27 (10)
O4—Fe—As1	138.90 (9)	O1^xvii^—K2—O3^iv^	119.27 (10)
O4^i^—Fe—As1	138.90 (9)	O1^vii^—K2—O3^iv^	114.43 (10)
O3^ii^—Fe—As1	90.12 (9)	O1^xviii^—K2—O3^iv^	65.57 (10)
O3^iii^—Fe—As1	90.12 (9)	O1—K2—O3^iv^	60.73 (10)
O1—Fe—As1	38.10 (8)	O3^xix^—K2—O3^iv^	180.0
O1^iv^—Fe—As1	38.09 (8)	O1^xvii^—K2—O3^xx^	65.57 (10)
O4—Fe—K2^iv^	145.08 (12)	O1^vii^—K2—O3^xx^	60.73 (10)
O4^i^—Fe—K2^iv^	86.44 (10)	O1^xviii^—K2—O3^xx^	119.27 (10)
O3^ii^—Fe—K2^iv^	53.01 (10)	O1—K2—O3^xx^	114.43 (10)
O3^iii^—Fe—K2^iv^	127.13 (9)	O3^xix^—K2—O3^xx^	79.69 (13)
O1—Fe—K2^iv^	88.80 (9)	O3^iv^—K2—O3^xx^	100.31 (13)
O1^iv^—Fe—K2^iv^	39.59 (9)	O1^xvii^—K2—O3^iii^	114.43 (10)
As1—Fe—K2^iv^	59.807 (14)	O1^vii^—K2—O3^iii^	119.27 (10)
O4—Fe—K2	86.44 (10)	O1^xviii^—K2—O3^iii^	60.73 (10)
O4^i^—Fe—K2	145.08 (12)	O1—K2—O3^iii^	65.57 (10)
O3^ii^—Fe—K2	127.13 (9)	O3^xix^—K2—O3^iii^	100.31 (13)
O3^iii^—Fe—K2	53.01 (10)	O3^iv^—K2—O3^iii^	79.69 (13)
O1—Fe—K2	39.59 (9)	O3^xx^—K2—O3^iii^	180.0
O1^iv^—Fe—K2	88.80 (9)	O1^xvii^—K2—As1^xvii^	30.70 (7)
As1—Fe—K2	59.807 (14)	O1^vii^—K2—As1^xvii^	84.61 (7)
K2^iv^—Fe—K2	119.61 (3)	O1^xviii^—K2—As1^xvii^	95.39 (7)
O4—Fe—K1^v^	44.10 (12)	O1—K2—As1^xvii^	149.30 (7)
O4^i^—Fe—K1^v^	96.21 (11)	O3^xix^—K2—As1^xvii^	30.21 (6)
O3^ii^—Fe—K1^v^	135.79 (11)	O3^iv^—K2—As1^xvii^	149.79 (6)
O3^iii^—Fe—K1^v^	44.07 (9)	O3^xx^—K2—As1^xvii^	67.56 (7)
O1—Fe—K1^v^	88.72 (9)	O3^iii^—K2—As1^xvii^	112.44 (7)
O1^iv^—Fe—K1^v^	131.25 (10)	O1^xvii^—K2—As1^xx^	84.61 (7)
As1—Fe—K1^v^	113.876 (15)	O1^vii^—K2—As1^xx^	30.70 (7)
K2^iv^—Fe—K1^v^	170.81 (3)	O1^xviii^—K2—As1^xx^	149.30 (7)
K2—Fe—K1^v^	54.545 (14)	O1—K2—As1^xx^	95.39 (7)
O4—Fe—K1^vi^	96.21 (11)	O3^xix^—K2—As1^xx^	67.56 (7)
O4^i^—Fe—K1^vi^	44.10 (12)	O3^iv^—K2—As1^xx^	112.44 (7)
O3^ii^—Fe—K1^vi^	44.07 (9)	O3^xx^—K2—As1^xx^	30.21 (6)
O3^iii^—Fe—K1^vi^	135.79 (11)	O3^iii^—K2—As1^xx^	149.79 (6)
O1—Fe—K1^vi^	131.25 (10)	As1^xvii^—K2—As1^xx^	71.54 (2)
O1^iv^—Fe—K1^vi^	88.72 (9)	O1^xvii^—K2—As1^iii^	95.39 (7)
As1—Fe—K1^vi^	113.876 (15)	O1^vii^—K2—As1^iii^	149.30 (7)
K2^iv^—Fe—K1^vi^	54.545 (15)	O1^xviii^—K2—As1^iii^	30.70 (7)
K2—Fe—K1^vi^	170.81 (3)	O1—K2—As1^iii^	84.61 (7)
K1^v^—Fe—K1^vi^	132.25 (3)	O3^xix^—K2—As1^iii^	112.44 (7)
O1—Ni—O1^vii^	91.64 (18)	O3^iv^—K2—As1^iii^	67.56 (7)
O1—Ni—O1^ii^	88.36 (18)	O3^xx^—K2—As1^iii^	149.79 (6)
O1^vii^—Ni—O1^ii^	180.0	O3^iii^—K2—As1^iii^	30.21 (6)
O1—Ni—O1^viii^	180.0	As1^xvii^—K2—As1^iii^	108.46 (2)
O1^vii^—Ni—O1^viii^	88.36 (18)	As1^xx^—K2—As1^iii^	180.00 (2)
O1^ii^—Ni—O1^viii^	91.64 (18)	O1^xvii^—K2—As1	149.30 (7)
O1—Ni—O2	92.81 (13)	O1^vii^—K2—As1	95.39 (7)
O1^vii^—Ni—O2	92.81 (13)	O1^xviii^—K2—As1	84.61 (7)
O1^ii^—Ni—O2	87.19 (13)	O1—K2—As1	30.70 (7)
O1^viii^—Ni—O2	87.19 (13)	O3^xix^—K2—As1	149.79 (6)
O1—Ni—O2^viii^	87.19 (13)	O3^iv^—K2—As1	30.21 (6)
O1^vii^—Ni—O2^viii^	87.19 (13)	O3^xx^—K2—As1	112.44 (7)
O1^ii^—Ni—O2^viii^	92.81 (13)	O3^iii^—K2—As1	67.56 (7)
O1^viii^—Ni—O2^viii^	92.81 (13)	As1^xvii^—K2—As1	180.0
O2—Ni—O2^viii^	180.0	As1^xx^—K2—As1	108.46 (2)
O1—Ni—K2^ix^	133.53 (9)	As1^iii^—K2—As1	71.54 (2)
O1^vii^—Ni—K2^ix^	133.53 (9)	O3^iv^—As1—O3	115.7 (2)
O1^ii^—Ni—K2^ix^	46.47 (9)	O3^iv^—As1—O1^iv^	114.24 (16)
O1^viii^—Ni—K2^ix^	46.47 (9)	O3—As1—O1^iv^	108.02 (17)
O2—Ni—K2^ix^	77.21 (12)	O3^iv^—As1—O1	108.02 (17)
O2^viii^—Ni—K2^ix^	102.79 (12)	O3—As1—O1	114.24 (16)
O1—Ni—K2	46.47 (9)	O1^iv^—As1—O1	94.9 (2)
O1^vii^—Ni—K2	46.47 (9)	O3^iv^—As1—Fe	122.14 (12)
O1^ii^—Ni—K2	133.53 (9)	O3—As1—Fe	122.14 (12)
O1^viii^—Ni—K2	133.53 (9)	O1^iv^—As1—Fe	47.43 (10)
O2—Ni—K2	102.79 (12)	O1—As1—Fe	47.43 (10)
O2^viii^—Ni—K2	77.21 (12)	O3^iv^—As1—K2	62.21 (12)
K2^ix^—Ni—K2	180.0	O3—As1—K2	140.81 (12)
O3—K1—O3^x^	171.66 (16)	O1^iv^—As1—K2	107.41 (11)
O3—K1—O3^xi^	110.90 (15)	O1—As1—K2	46.24 (11)
O3^x^—K1—O3^xi^	68.45 (15)	Fe—As1—K2	73.134 (11)
O3—K1—O3^xii^	68.45 (15)	O3^iv^—As1—K2^iv^	140.80 (12)
O3^x^—K1—O3^xii^	110.90 (15)	O3—As1—K2^iv^	62.21 (12)
O3^xi^—K1—O3^xii^	171.66 (16)	O1^iv^—As1—K2^iv^	46.23 (11)
O3—K1—O4^xiii^	110.09 (12)	O1—As1—K2^iv^	107.41 (11)
O3^x^—K1—O4^xiii^	61.98 (9)	Fe—As1—K2^iv^	73.134 (11)
O3^xi^—K1—O4^xiii^	61.98 (9)	K2—As1—K2^iv^	146.27 (2)
O3^xii^—K1—O4^xiii^	110.09 (12)	O3^iv^—As1—K1	95.04 (13)
O3—K1—O4^xiv^	61.98 (9)	O3—As1—K1	39.86 (12)
O3^x^—K1—O4^xiv^	110.09 (12)	O1^iv^—As1—K1	88.62 (11)
O3^xi^—K1—O4^xiv^	110.09 (12)	O1—As1—K1	152.66 (11)
O3^xii^—K1—O4^xiv^	61.98 (9)	Fe—As1—K1	129.701 (14)
O4^xiii^—K1—O4^xiv^	59.17 (17)	K2—As1—K1	155.88 (2)
O3—K1—O2^xv^	77.16 (9)	K2^iv^—As1—K1	57.180 (19)
O3^x^—K1—O2^xv^	110.34 (10)	O3^iv^—As1—K1^xxi^	39.86 (12)
O3^xi^—K1—O2^xv^	77.16 (9)	O3—As1—K1^xxi^	95.04 (13)
O3^xii^—K1—O2^xv^	110.34 (10)	O1^iv^—As1—K1^xxi^	152.66 (11)
O4^xiii^—K1—O2^xv^	138.57 (8)	O1—As1—K1^xxi^	88.62 (11)
O4^xiv^—K1—O2^xv^	138.57 (8)	Fe—As1—K1^xxi^	129.701 (14)
O3—K1—O2^xvi^	110.34 (10)	K2—As1—K1^xxi^	57.180 (18)
O3^x^—K1—O2^xvi^	77.16 (9)	K2^iv^—As1—K1^xxi^	155.88 (2)
O3^xi^—K1—O2^xvi^	110.34 (10)	K1—As1—K1^xxi^	100.60 (3)
O3^xii^—K1—O2^xvi^	77.16 (9)	O2^xxii^—As2—O2	112.4 (3)
O4^xiii^—K1—O2^xvi^	138.57 (8)	O2^xxii^—As2—O4	109.74 (11)
O4^xiv^—K1—O2^xvi^	138.57 (8)	O2—As2—O4	109.74 (11)
O2^xv^—K1—O2^xvi^	60.88 (19)	O2^xxii^—As2—O4^xxiii^	109.74 (11)
O3—K1—As2^xvi^	94.17 (8)	O2—As2—O4^xxiii^	109.74 (11)
O3^x^—K1—As2^xvi^	94.17 (8)	O4—As2—O4^xxiii^	105.2 (3)
O3^xi^—K1—As2^xvi^	94.17 (8)	O2^xxii^—As2—K1^xxiv^	56.20 (15)
O3^xii^—K1—As2^xvi^	94.17 (8)	O2—As2—K1^xxiv^	56.20 (15)
O4^xiii^—K1—As2^xvi^	150.41 (9)	O4—As2—K1^xxiv^	127.38 (15)
O4^xiv^—K1—As2^xvi^	150.41 (9)	O4^xxiii^—As2—K1^xxiv^	127.38 (15)
O2^xv^—K1—As2^xvi^	30.44 (9)	O2^xxii^—As2—K1^v^	123.80 (15)
O2^xvi^—K1—As2^xvi^	30.44 (9)	O2—As2—K1^v^	123.80 (15)
O3—K1—As2^xiii^	85.83 (8)	O4—As2—K1^v^	52.62 (15)
O3^x^—K1—As2^xiii^	85.83 (8)	O4^xxiii^—As2—K1^v^	52.62 (15)
O3^xi^—K1—As2^xiii^	85.83 (8)	K1^xxiv^—As2—K1^v^	180.0
O3^xii^—K1—As2^xiii^	85.83 (8)	As1—O1—Ni	123.99 (17)
O4^xiii^—K1—As2^xiii^	29.59 (9)	As1—O1—Fe	94.47 (13)
O4^xiv^—K1—As2^xiii^	29.59 (9)	Ni—O1—Fe	127.73 (17)
O2^xv^—K1—As2^xiii^	149.56 (9)	As1—O1—K2	103.06 (15)
O2^xvi^—K1—As2^xiii^	149.56 (9)	Ni—O1—K2	97.08 (12)
As2^xvi^—K1—As2^xiii^	180.0	Fe—O1—K2	107.88 (13)
O3—K1—K2^xiii^	122.97 (7)	As2—O2—Ni	133.4 (2)
O3^x^—K1—K2^xiii^	55.47 (7)	As2—O2—K1^xxiv^	93.36 (19)
O3^xi^—K1—K2^xiii^	122.97 (7)	Ni—O2—K1^xxiv^	133.23 (19)
O3^xii^—K1—K2^xiii^	55.47 (7)	As1—O3—Fe^xiv^	136.97 (19)
O4^xiii^—K1—K2^xiii^	82.19 (3)	As1—O3—K1	117.12 (17)
O4^xiv^—K1—K2^xiii^	82.19 (3)	Fe^xiv^—O3—K1	104.24 (13)
O2^xv^—K1—K2^xiii^	129.43 (10)	As1—O3—K2^iv^	87.58 (13)
O2^xvi^—K1—K2^xiii^	68.55 (9)	Fe^xiv^—O3—K2^iv^	92.78 (12)
As2^xvi^—K1—K2^xiii^	98.99 (3)	K1—O3—K2^iv^	74.37 (9)
As2^xiii^—K1—K2^xiii^	81.01 (3)	As2—O4—Fe	124.58 (13)
O3—K1—K2^iv^	55.46 (7)	As2—O4—Fe^i^	124.58 (13)
O3^x^—K1—K2^iv^	122.97 (7)	Fe—O4—Fe^i^	97.79 (17)
O3^xi^—K1—K2^iv^	55.47 (7)	As2—O4—K1^v^	97.79 (18)
O3^xii^—K1—K2^iv^	122.97 (7)	Fe—O4—K1^v^	104.58 (14)
O4^xiii^—K1—K2^iv^	82.19 (3)	Fe^i^—O4—K1^v^	104.58 (14)
O4^xiv^—K1—K2^iv^	82.19 (3)		

Symmetry codes: (i) −*x*+1/2, −*y*+1/2, −*z*+3/2; (ii) −*x*, *y*, −*z*+1; (iii) *x*+1/2, −*y*+1/2, −*z*+1; (iv) −*x*+1/2, −*y*+1/2, *z*; (v) *x*+1/2, *y*+1/2, *z*+1/2; (vi) −*x*, −*y*, −*z*+1; (vii) *x*, −*y*+1, *z*; (viii) −*x*, −*y*+1, −*z*+1; (ix) *x*−1, *y*, *z*; (x) *x*, −*y*, −*z*+1/2; (xi) *x*, −*y*, *z*; (xii) *x*, *y*, −*z*+1/2; (xiii) *x*−1/2, *y*−1/2, *z*−1/2; (xiv) *x*−1/2, −*y*+1/2, −*z*+1; (xv) *x*+1/2, *y*−1/2, −*z*+1; (xvi) *x*+1/2, *y*−1/2, *z*−1/2; (xvii) −*x*+1, −*y*+1, −*z*+1; (xviii) −*x*+1, *y*, −*z*+1; (xix) *x*+1/2, *y*+1/2, −*z*+1; (xx) −*x*+1/2, *y*+1/2, *z*; (xxi) −*x*+1/2, −*y*+1/2, −*z*+1/2; (xxii) *x*, *y*, −*z*+3/2; (xxiii) *x*, −*y*+1, −*z*+3/2; (xxiv) *x*−1/2, *y*+1/2, *z*+1/2.

## References

[bb1] Adams, S. (2003). *softBV*. Université de Göttingen, Allemagne. http://kristall.uni-mki. gwdg.de/softBV/

[bb2] Alhakmi, G., Assani, A., Saadi, M. & El Ammari, L. (2013*a*). *Acta Cryst.* E**69**, i40. 10.1107/S1600536813016504 PMC3772398 24046541

[bb3] Alhakmi, G., Assani, A., Saadi, M., Follet, C. & El Ammari, L. (2013*b*). *Acta Cryst.* E**69**, i56. 10.1107/S1600536813020977 PMC3884376 24426976

[bb4] Arroyo-de Dompablo, M. E., Amador, U., Alvarez, M., Gallardo, J. & García-Alvarado, F. (2006). *Solid State Ionics*, **177**, 2625–2628.

[bb5] Assani, A., Saadi, M., Alhakmi, G., Houmadi, E. & El Ammari, L. (2013). *Acta Cryst.* E**69**, i60. 10.1107/S1600536813023106 PMC3884417 24426979

[bb6] Attfield, J. P., Battle, P. D., Cheetham, A. K. & Johnson, D. C. (1989). *Inorg. Chem.* **28**, 1207–1213.

[bb7] Barpanda, P., Liu, G., Ling, C. D., Tamaru, M., Avdeev, M., Chung, S. C., Yamada, Y. & Yamada, A. (2013). *Chem. Mater.* **25**, 3480–3487.

[bb8] Baur, W. H. (1974). *Acta Cryst.* B**30**, 1195–1215.

[bb9] Beneke, K. & Lagaly, G. (1982). *Clay Miner.* **17**, 175–183.

[bb11] Ben Smail, R. & Driss, A. (2007). *Acta Cryst.* A**63**, s277.

[bb12] Ben Smail, R., Driss, A. & Jouini, T. (1999). *Acta Cryst.* C**55**, 284–286.

[bb13] Ben Smail, R., Hlel, F. & Driss, A. (2007). 1^ière^ Conférence Internationale sur la Métallurgie et l’Environnement, Annaba, Algérie.

[bb14] Ben Smail, R. & Jouini, T. (2004). *Acta Cryst.* E**60**, i1–i2.

[bb10] Ben Smail, R. & Jouini, T. (2005). *Ann. Chim. Sci. Mat.* **30**, 119–132.

[bb15] Ben Smail, R. & Zid, M. F. (2017*a*). En cours de préparation.

[bb16] Ben Smail, R. & Zid, M. F. (2017*b*). En cours de préparation.

[bb17] Ben Smail, R., Zid, M. F. & Jouini, T. (2002). *J. Soc. Chim. Tunis.* **4**, 1665–1673.

[bb18] Bouraima, A., Makani, T., Assani, A., Saadi, M. & El Ammari, L. (2016). *Acta Cryst.* E**72**, 1143–1146. 10.1107/S2056989016011373 PMC4971858 27536399

[bb19] Bragg, W. L. & Brown, G. B. (1926). *Z. Kristallogr.* **63**, 538–556.

[bb20] Brandenburg, K. (2006). *DIAMOND*. Crystal Impact GbR, Bonn, Allemagne.

[bb21] Brown, I. D. (2002). *The Chemical Bond in Inorganic Chemistry – The Bond Valence Model*. IUCr Monographs on Crystallography, 12. Oxford University Press.

[bb22] Buckley, A. M., Bramwell, S. T., Day, P. & Harrison, W. T. A. (1988). *Z. Naturforsch. Teil B*, **43**, 1053–1055.

[bb23] Buckley, A. M., Bramwell, S. T., Visser, D. & Day, P. (1987). *J. Solid State Chem.* **69**, 240–251.

[bb24] Duisenberg, A. J. M. (1992). *J. Appl. Cryst.* **25**, 92–96.

[bb25] Essehli, R., Belharouak, I., Ben Yahia, H., Chamoun, R., Orayech, B., El Bali, B., Bouziane, K., Zhou, X. L. & Zhou, Z. (2015). *Dalton Trans.* **44**, 4526–4532. 10.1039/c5dt00021a 25652612

[bb26] Farrugia, L. J. (2012). *J. Appl. Cryst.* **45**, 849–854.

[bb27] Glaum, R., Gruehn, R. & Möller, M. (1986). *Z. Anorg. Allg. Chem.* **543**, 111–116.

[bb28] Harms, K. & Wocadlo, S. (1995). *XCAD4*. University of Marburg, Allemagne.

[bb29] Harris, F. E. & Monkhorst, H. J. (1970). *Phys. Rev. B*, **2**, 4400–4405.

[bb30] Hidouri, M., Lajmi, B., Driss, A. & Ben Amara, M. (2004). *J. Chem. Crystallogr.* **34**, 669–672.

[bb31] Hoppe, R. (1966). *Angew. Chem. Int. Ed. Engl.* **5**, 95–106.

[bb32] Hoppe, R. (1970). *Angew. Chem. Int. Ed. Engl.* **9**, 25–34.

[bb33] Jansen, M. (1978). *Z. Anorg. Allg. Chem.* **441**, 5–12.

[bb34] Jones, P. G., Range, K. J. & Meister, H. (1987). *Z. Naturforsch. Teil B*, **42**, 1365–1366.

[bb35] Kinomura, N., Matsui, N., Kumada, N. & Muto, F. J. (1989). *J. Solid State Chem.* **79**, 232–237.

[bb36] Korzenski, M. B., Kolis, J. W. & Long, G. J. (1999). *J. Solid State Chem.* **147**, 390–398.

[bb37] Macíček, J. & Yordanov, A. (1992). *J. Appl. Cryst.* **25**, 73–80.

[bb38] Malingowski, A. C., Stephens, P. W., Huq, A., Huang, Q., Khalid, S. & Khalifah, P. G. (2012). *Inorg. Chem.* **51**, 6096–6103. 10.1021/ic202715c 22530995

[bb39] Marzouki, R., Frigui, W., Guesmi, A., Zid, M. F. & Driss, A. (2013). *Acta Cryst.* E**69**, i65–i66. 10.1107/S1600536813025233 PMC3790335 24098157

[bb40] Masquelier, C., Padhi, A. K., Nanjundaswamy, K. S. & Goodenough, J. B. (1998). *J. Solid State Chem.* **135**, 228–234.

[bb41] Mesa, J. L., Pizarro, J. L., Lezama, L., Escobal, J., Arriortua, M. I. & Rojo, T. (1998). *J. Solid State Chem.* **141**, 508–513.

[bb42] Momma, K. & Izumi, F. (2008). *J. Appl. Cryst.* **41**, 653–658.

[bb43] Nespolo, M. (2015). *CHARDI-2015.* http://www.crystallography.fr/chardi.

[bb44] Nespolo, M. (2016). *Acta Cryst.* B**72**, 51–66. 10.1107/S2052520615019472 26830796

[bb45] Nespolo, M., Ferraris, G., Ivaldi, G. & Hoppe, R. (2001). *Acta Cryst.* B**57**, 652–664. 10.1107/s0108768101009879 11574721

[bb46] Nespolo, M. & Guillot, B. (2016). *J. Appl. Cryst.* **49**, 317–321.

[bb47] North, A. C. T., Phillips, D. C. & Mathews, F. S. (1968). *Acta Cryst.* A**24**, 351–359.

[bb48] Nose, M., Nakayama, H., Nobuhara, K., Yamaguchi, H., Nakanishi, S. & Iba, H. (2013). *J. Power Sources*, **234**, 175–179.

[bb49] Ouaatta, S., Assani, A., Saadi, M. & El Ammari, L. (2015). *Acta Cryst.* E**71**, 1255–1258. 10.1107/S205698901501779X PMC4647370 26594419

[bb50] Rittner, P. & Glaum, R. (1994). *Z. Kristallogr.* **209**, 162–169.

[bb51] Rochère, M. de la, Kahn, A., d’Yvoire, F. & Bretey, E. (1985). *Mater. Res. Bull.* **20**, 27–34.

[bb52] Sanz, F., Parada, C., Rojo, J. M. & Ruiz-Valero, C. (1999). *Chem. Mater.* **11**, 2673–2679.

[bb53] Sheldrick, G. M. (2008). *Acta Cryst.* A**64**, 112–122. 10.1107/S0108767307043930 18156677

[bb54] Sheldrick, G. M. (2015). *Acta Cryst.* C**71**, 3–8.

[bb55] Souiwa, K., Chennabasappa, M., Decourt, R., Ben Amara, M., Hidouri, M. & Toulemonde, O. (2015*a*). *Inorg. Chem.* **54**, 7345–7352. 10.1021/acs.inorgchem.5b00776 26161799

[bb56] Souiwa, K., Hidouri, M., Toulemonde, O., Duttine, M. & Ben Amara, M. (2015*b*). *J. Alloys Compd.* **627**, 153–160.

[bb57] Westrip, S. P. (2010). *J. Appl. Cryst.* **43**, 920–925.

[bb58] Wildner, M. (1992). *Z. Kristallogr.* **202**, 51–70.

[bb59] Yahia, H. B., Essehli, R., Avdeev, M., Park, J. B., Sun, Y. K., Al-Maadeed, M. A. & Belharouak, I. (2016). *J. Solid State Chem.* **238**, 103–108.

[bb60] Zachariasen, W. H. (1928). *Skr. Norske Vidensk. Akad. Oslo I Mat. Naturv. Kl.* pp. 1–165.

[bb61] Zintl, E., Harder, A. & Dauth, B. (1934). *Z. Elektrochem.* **40**, 588–593.

